# Chemopreventive Effects and Antioxidant Capacity of Combined Leaf Extracts of *Sesamum angustifolium* (Oliv.) Engl. and *Hibiscus articulatus* on Rhabdomyosarcoma

**DOI:** 10.1155/2020/8567182

**Published:** 2020-03-26

**Authors:** Clayton E. Siamayuwa, Loveness K. Nyanga, Cathrine Chidewe

**Affiliations:** ^1^Department of Biochemistry, University of Zimbabwe, P.O. Box MP 167 Mount Pleasant, Harare, Zimbabwe; ^2^Institute of Food and Nutritional Sciences, University of Zimbabwe, P.O. Box MP 167 Mount Pleasant, Harare, Zimbabwe

## Abstract

*Sesamum angustifolium* (Oliv.) Engl. and *Hibiscus articulatus* contain compounds that have antimutagenic properties. The rise in rhabdomyosarcoma in paediatrics and prognosis of the disease in infants compared to adults calls for newer, less toxic alternatives in treatment of the disease. The aim of this study was to determine the anticancer activity and antioxidant capacity of combined leaf extracts of *Sesamum angustifolium* (Oliv.) Engl. and *Hibiscus articulatus* (SAHA), against rhabdomyosarcoma (RMS) using rhabdomyosarcoma (RD) cell line and mouse (L20B) cell line. Cytotoxicity, morphology, apoptosis induction, and antioxidant capacity assays were done. Of the four solvents used for extraction, the dichloromethane SAHA extract was the most cytotoxic with IC_50_ of 106 *μ*g/mL after doxorubicin, the reference anticancer drug with IC_50_ of 0.8 *μ*g/mL. The SAHA extracts had a stronger cytotoxicity effect on the cancerous RD cells than on normal L20B cells. Morphological assessment showed untreated cells maintained their normal striated appearance of muscle cells whereas cells treated with doxorubicin or SAHA extracts exhibited cell shrinkage, loss of surface adherence, reduced cell density along with cell debris, which is a characteristic of apoptosis. Normal L20B cells when treated with doxorubicin or SAHA extracts, maintained their cell shape, and remained adherent to the surface. The apoptotic enzyme caspase-3 was induced in a concentration dependent manner upon treatment of the RD cells with SAHA extracts or doxorubicin. Induction of caspase-3 was ten times less in treated L20B cells compared to the RD cells. Low induction of caspase-9 enzyme was observed in both treated RD and L20B cells. Treatment of both RD and L20B cells with SAHA extracts or doxorubicin resulted in increased activity of peroxidase and reduction of oxidative stress. Results of the study show that the SAHA extracts are potential sources of compounds that may serve as useful agents for treatment of rhabdomyosarcoma.

## 1. Introduction

Cancer is the third leading cause of death in the world. In 2018, the cancer morbidity rate was 18.1 million and mortality rate was 9.6 million [1]. The annual cancer mortality and morbidity rates are likely to increase in the near future. The World Health Organization projected an annual increase to 26 million new cases and 17 million deaths by 2030, with developing countries bearing the heaviest burden [2].

Rhabdomyosarcoma is listed among the top 20 most diagnosed cancers in the world. RMS is a soft tissue neoplasm that share a propensity to undergo myogenesis [3]. RMS is the third most common childhood solid tumor after neuroblastoma and Wilms' tumor accounting for 4–6% of all paediatric tumors [4, 5]. About 350 new cases of RMS are diagnosed yearly in the United States and 60 new cases in the UK [5]. In Southern Africa, rhabdomyosarcoma and other soft tissue sarcomas constitute 70% in Kenya, 58% in Mozambique, and 21% in Zimbabwe of all paediatric cancers according to 2010–2012 statistics [6].

The survival rate of a patient diagnosed of RMS is 50–70% [7]. The survival rate has been improved by the available clinical treatment methods which include laser surgery, radiation therapy, chemotherapy, organ sparing, and transoral robotic surgery [8]. Some of the current therapies for the treatment of RMS are associated with high expense, increased toxicity, and numerous side effects [5].

Medicinal plants have been proposed as an alternate source of medicament, since they have been used for centuries [9]. Medicinal plants are rich in chemical substances in the form of secondary metabolites or phytochemicals. The mechanism of action of the phytochemicals in cancer treatment is drug-like and biologically friendly compared to synthetic molecules [10]. Phytochemicals have been reported to possess the ability to control cell growth in cancerous cells via the deregulation of cell cycle proteins, thereby preventing the abnormal cells from proliferating [11]. Phytochemicals such as triterpenes, flavonoids, and polyphenols have shown the ability to upregulate apoptotic proteins, caspase 3/9, involved in an active form of programmed cell death called apoptosis [12–14].

*Sesamum angustifolium* (Oliv.) Engl. is a perennial wild vegetable native to tropical and subtropical Africa. The mucilage of rubbed leaves in water is traditionally used to treat eye troubles, burns, wounds, and diarrhea in children while the crushed leaves can be used as a soap substitute due to their mucilaginous nature [15]. *Hibiscus articulatus* is an erect semiwoody herb native to west and tropical Africa [16]. The leaves of *Hibiscus articulatus* are of great economic importance as a source of medicinal products, food, and cosmetics.

The aim of the study was to evaluate the combined effect of SAHA extracts on RD cancer cells and L20B normal cells in relation to the reduction of high levels of oxidative stress, cytotoxicity, and induction of apoptosis.

## 2. Materials and Methods

### 2.1. Chemicals

Chemicals, media, and drugs used were purchased from Sigma-Aldrich (Steinheim, Germany) and were of analytical grade, these include RD cells; L20B cells; eagle's minimum essential medium (MEM), Fetal Bovine Serum (FBS), Gibco Phosphate-Buffered Saline (GPBS) without Ca^2+^ and Mg^2+^ (life technologies), Penicillin/Streptomycin, trypsin/EDTA, doxorubicin, propidium iodide (PI), Thiazolyl Blue Tetrazolium Bromide (MTT) and dimethyl sulphoxide (DMSO).

### 2.2. Preparation of Plant Material

*Sesamum angustifolium* (Oliv.) Engl. and *Hibiscus articulatus* were collected from two tree farms in Zimbabwe ((17°23′S, 30°24′E), in the month of December. The plants were identified and authenticated by a botanist from the Botanical gardens of Zimbabwe. Voucher specimens of the plants were deposited in the national herbarium, *Sesamum angustifolium* (Oliv.) Engl. voucher specimen number (68726) and *Hibiscus articulatus*, voucher specimen number (68728). Young leaves from aerial parts of the plants were air-dried and pulverized into powder using a laboratory homogeniser (Christy and Norris Ltd. Chemsford, England).

### 2.3. Preparation of Extracts

The powdered samples (25 g) were subjected to exhaustive solvent extraction for 8 hours using a Soxhlet apparatus and 250 mLs each of the solvents dichloromethane, acetone, and methanol successively. After extraction with methanol, the plant residue was extracted with water at 25°C for 8 hrs with continuous shaking. The dichloromethane, acetone, and methanol fractions were concentrated by rotary evaporation in a Buchi R205 rotary evaporator (Buchi, Switzerland) at 40°C. The aqueous extract was freeze-dried on a Christ Alpha 1–4 freeze drier (Dorfen, Germany). To prepare the combined (SAHA) extract solution for testing, equal amounts of the dried extracts of *Sesamum angustifolium* (Oliv.) Engl. and *Hibiscus articulatus* were weighed, mixed, and dissolved in appropriate amount of solvent to achieve the desired concentration. The combined extracts were dissolved in dimethyl sulphoxide (DMSO) and Eagle's minimum essential medium to a final concentration of 0.65% DMSO.

### 2.4. Proliferation of RD and L20B Cells

The human rhabdomyosarcoma (RD) cancer cell line and the mouse (LB20) normal cell line were cultured in a humid environment at 37°C and 5% CO_2_ in Eagle's minimum essential medium supplemented with 10% fetal bovine serum and 1% streptomycin/penicillin. At 90% confluence, the cells were harvested using 0.25% trypsin/0.53 mM EDTA solution (Sigma-Aldrich, USA) and subcultured onto 96 well plates.

### 2.5. Determination of Cytotoxicity

The cytotoxity assay (MTT assay) was carried out following the procedure previously described by [17] with modifications. The trypsinized RD and L20B cells were seeded in a 96 well plate at a density of 5 × 10^4^ cells per well. After 48 hrs incubation, the medium was removed from each well and 300 *μ*L of fresh medium added. One hundred microliter of 400 *μ*g/ml crude extract was serially diluted at concentrations ranging from 200–12.5 *μ*g/mL in rows D to H of the microtiter plate. Row A served as a blank (containing medium only) while row B served as a positive control (untreated cells). Row C contained untreated cells in 0.65% DMSO. The cells with and without extract were incubated in a Biobase QP 160-II CO_2_ incubator at 37°C for 48 hrs before determining cell viability (Biobase, China). The test was performed in triplicate.

After incubation, the culture medium with or without extract was removed from the plates and replaced with 100 *μ*L fresh culture medium. Five microliters of 0.5 mg/ml of MTT, 3-(4,5-Dimethylthiazol-2-yl)-2,5-Diphenyltetrazolium bromide, in PBS were added to each well. After 2 hr incubation at 37°C, the medium with MTT was removed and 100 *μ*L of DMSO added to dissolve the formed formazan crystals. The cells were incubated for 30 mins and optical density measured on an ELISA reader (Biobase, China) at 495 nm. Percentage cell viability was calculated as follows:

  % cell viability = (mean absorbance of sample − blank/mean absorbance of control-blank) × 100

### 2.6. Morphological Assessment

The confluent RD and L20B cells in 24 well plates were treated with various concentrations (IC_50_) of each SAHA extract and doxorubicin (0.8 *μ*g/mL) for 48 hrs and morphological alterations observed under an inverted microscope (Leitz, Germany) at 400x magnification and photographs were taken using a Japson MD130 digital camera (Japson, China). The pictures were analyzed using a Future Win Joe software for windows.

### 2.7. Determination of Apoptotic Induction Using PI Assay

The effect of SAHA leaf extracts on RD cells and L20B cells was analyzed using propidium iodide following the method described by [18], with some modifications. PI is capable of passing through damaged cell membranes, and binding to DNA. RD and L20B cells were harvested at exponential phase of growth and seeded into 16 well plates at a seeding density of 1 × 10^4^ viable cells/well. After 48 hr incubation at 37°C, an aliquot of 100 *μ*l of SAHA extracts (50 *μ*g/mL and 200 *μ*g/mL) and doxorubicin (2 *μ*g/mL and 8 *μ*g/mL) were added to the wells in triplicate. Control wells containing untreated cells were also included. The plates were incubated for 48 hours after which the media were aspirated from the wells. The cells were resuspended in 250 *μ*L of 1X annexin V binding buffer and 5 *μ*L of propidium iodide were added. The cells were placed in the dark for 15 minutes. The absorbance was read under an FMAX374 fluorescence microplate spectrophotometer (Molecular Devices, Sunnyvale, USA) at an excitation wavelength of 544 nm and an emission wavelength of 620 nm. The plates were frozen to kill all the cells and then thawed before fluorescence was measured as before. Growth stimulation/inhibition was calculated as *T*/*C* × 100%, where *T* and *C* are the fluorescence readings of the test and control samples, respectively. *T*/*C* greater than 125% indicated stimulation while *T*/*C* <30% indicated cytotoxicity [18].

### 2.8. Caspase 3 and Caspase 9 Activity Assays

Caspase 3 or caspase 9 colorimetric assay was conducted as per manufacturer's instructions (Abcam, UK). RD and L20B cells were treated with different concentrations of SAHA extracts of 50, 100, and 200 *μ*g/mL and doxorubicin, 2, 4, and 8 *μ*g/mL for 24 hours. The cells were harvested and pelleted at a concentration of 2–5 × 10^6^ cells/mL. The cells were resuspended in 50 *μ*L lysis buffer and protein concentration determined using the BCA assay. The protein was diluted to achieve a concentration of 50–200 *μ*g protein per 50 *μ*L lysis buffer per each well. To each well was added 50 *μ*L of cytosolic extract and 50 *μ*L of 2X reaction buffer containing 10 mM Dithiothreitol (DTT). A control well was included containing cytosolic extract from untreated cells and background well containing 50 *μ*L of reaction buffer (with 10 mM DTT). An aliquot of 5 *μ*L of DEVD-p-NA (caspase 3 substrate) or LEHD-p-NA (caspase 9 substrate) was added and the mixture incubated for 60 minutes at 37°C in a CO_2_ incubator (Biobase, China). After incubation, the absorbance was read at 405 nm in an ELISA reader (Biobase, China). Background reading was subtracted from the samples before calculating fold increase. Fold increase in caspase 3 and caspase 9 was determined by comparing treated sample results to the level of untreated control.

### 2.9. Antioxidant Capacity of Treated Cells

#### 2.9.1. Peroxidase Assay

The peroxidase assay was done using a peroxidase assay kit (KPL Inc., Germany) following the manufacturer's recommendations with some modifications. Treated and untreated RD and L20B cells were subjected to lysis buffer and the protein concentration was determined. An aliquot (10 *μ*L) of sample or standard was added to 96 well plate followed by 170 *μ*L of 50 mM phosphate buffer, pH 7.0. An aliquot of 50 *μ*L microwell peroxidase substrate was added into each well and mixed. The reaction was initiated by adding 75 *μ*L of hydrogen peroxide working solution, prepared by mixing 10 mL of 50 mM phosphate buffer and 34 *μ*L of 30% v/v of hydrogen peroxide. The reaction was done in duplicate. Absorbance was read at 620 nm at 75 second intervals. Peroxidase activity was calculated using the equation obtained from linear regression of the standard curve.

#### 2.9.2. Thiobarbituric Acid Reacting Substance (TBARS) Assay

This assay was used to monitor lipid peroxidation which is a major indicator of oxidative stress. TBARS are expressed in terms of malonaldehyde (MDA) equivalents; as such MDA standard was used to construct a standard curve. Treated and untreated RD and L20B cells were subjected to lysis buffer and protein concentration determined. An aliquot (12.6 *μ*L) of 100% ethanol was added to 100 *μ*L of supernatant in a tube, followed by 100 *μ*L of orthophosphoric acid. The mixture was vortexed for 10 seconds after which 12.5 *μ*L of thiobarbituric acid (TBA) reagent (0.11 M in 0.1 M sodium hydroxide) were added. The mixture was further mixed for 10 seconds and heated in a 90°C water bath for 45 minutes. The mixture was transferred to ice for 2 minutes in order to stop the reaction. The mixture was transferred to room temperature for 5 minutes, after which 1000 *μ*L of *n*-butanol and 100 *μ*L of 0.14 M NaCI were added. The mixture was then vortexed for 10 seconds. The samples were centrifuged at 12,000 ×g for 2 minutes at 4°C. A volume (250 *μ*L) of the top butanol phase was added into each well of the 96 well plate. The reaction was done in triplicate. The absorbance was read at 532 nm on a microplate reader (Biobase, China). TBARS results were expressed in nmoles/mg protein.

### 2.10. Statistical Analysis

Statistical analysis was carried out with GraphPad Prism (Graphpad Software Inc., San Diago, California, USA) version 5.0 using ANOVA followed by Turkey's test (*p* values < 0.05 regarded as significant).

## 3. Results

### 3.1. Yield of Extracts

The yield of phytochemicals increased with increase in solvent polarity, as shown in Table 1. The aqueous fractions had the highest percentage yield of phytochemicals followed by methanol, acetone, and dichloromethane fractions in both *Sesamum angustifolium* (Oliv.) Engl. and *Hibiscus articulatus* leaves. The aqueous fraction of both *Sesamum angustifolium* (Oliv.) Engl. and *Hibiscus articulatus* had similar yield of 23%. There was no significant difference (*p* > 0.05) in the yield of the methanolic fractions of *Hibiscus articulatus* (20.5 ± 1.0%) and *Sesamum angustifolium* (Oliv.) Engl. (18.5 ± 1.1%) respectively. The yield of dichloromethane fractions of both *Sesamum angustifolium* (Oliv.) Engl. (7.1 ± 3.1%) and *Hibiscus articulatus* (7.1 ± 2.0%) was similar. The yield of the acetone extracts was also similar for both *S. angustifolium* (Oliv.) Engl. (16.7 ± 1.4) and *H. articulatus* (16.9 ± 2.3).

### 3.2. Determination of Cytotoxicity

As shown in Table 2, the cell viability in both RD and L20B cells decreased with the increase in the concentration of extract. L20B cells had higher cell viability compared to RD cells for all treatments. Doxorubicin recorded significantly lower cell viability for RD cells compared to all SAHA extracts at all concentrations used.

Doxorubicin was used as a reference anticancer drug. It had the highest cytotoxicity effect on RD cells (IC_50_ = 0.8 *μ*g/mL), compared to SAHA fractions (Table 3). Among the SAHA fractions, dichloromethane extract had the highest cytotoxicity effect on RD cells with IC_50_ value of 106 *μ*g/mL followed by methanol extract (IC_50_ = 122 *μ*g/mL), aqueous extract (IC_50_ = 129 *μ*g/mL), and acetone (IC_50_ = 158 *μ*g/mL).

### 3.3. Determination of the Effect of SAHA Extracts on Cell Morphology

**Figure 1:**
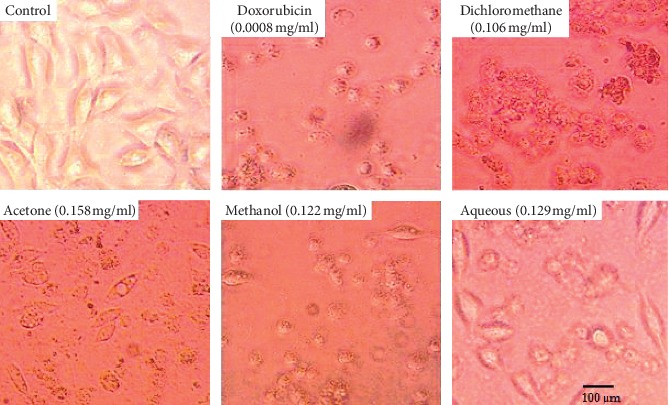
Morphological alterations on RD cells at IC_50_ of SAHA extracts and doxorubicin. Photomicrographs taken at 400x magnification using a Japson MD130 digital camera. The pictures were analyzed using a future win joe software for windows. Untreated RD cells (control) appeared in normal shape with 95–100% confluence. RD cells treated with SAHA extracts and doxorubicin showed loss of cell adherence, shrinking of cells, and reduced cell density along with cell debris.

**Figure 2:**
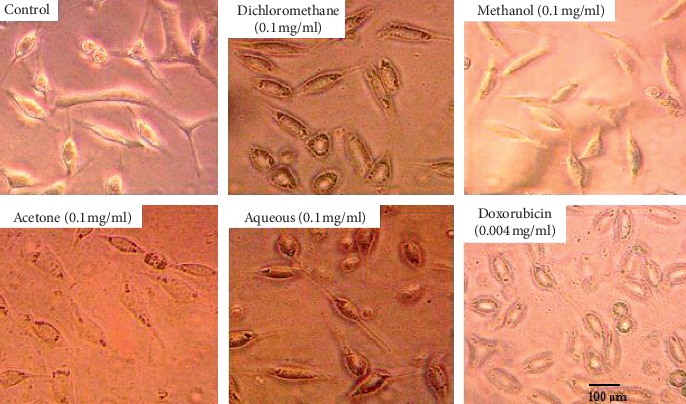
Morphological alterations on L20B cells at IC_50_ of SAHA extracts and doxorubicin. Photomicrographs taken at 400x magnification using a Japson MD130 digital camera. The pictures were analyzed using a future win joe software for windows. Untreated (control) and treated L20B cells appeared in normal shape with 50–75% confluence. No morphological alterations, characteristic of apoptosis, were observed in the treated cells.

The effect of SAHA extracts and doxorubicin on RD and L20B cell morphology is shown in Figures 1 and 2 respectively. Both untreated RD and L20B cells appeared in normal shape with 70–100% confluence. Treated RD cells showed loss of cell adherence, shrinking of cells, and reduced cell density along with cell debris (Figure 1). The alteration in cell morphology was intense in cells treated with doxorubicin, dichloromethane extract, and methanol extract followed by acetone extract and lastly aqueous extract. The morphological alterations seemed to follow the IC_50_ trend observed. Treated L20B cells did not have any loss of cell adherence, shrinking of cells, or reduced cell density as shown in Figure 2. The treated cells appeared normal in shape with 50–70% confluence.

### 3.4. Determination of the Effect of SAHA Extracts Using Propidium Iodide

As shown in Table 4, cytotoxicity induction was observed in both SAHA extracts and doxorubicin (T/C% range 93%–48%). Of the extracts, the highest apoptotic induction effect of T/C 48.4 + 2.7%, was observed in RD cells treated with 200 *μ*g/mL methanol. Aqueous extract (50 *μ*g/mL) recorded the lowest cytotoxicity effect on RD cells of *T*/*C* 93.0 + 10.6%. A significant apoptotic induction effect was observed in RD cells treated with 200 *μ*g/mL of all SAHA extracts compared to treated L20B cells.

### 3.5. Induction Effects of SAHA Extracts on Caspase-3 and Caspase-9 Activity

**Figure 3:**
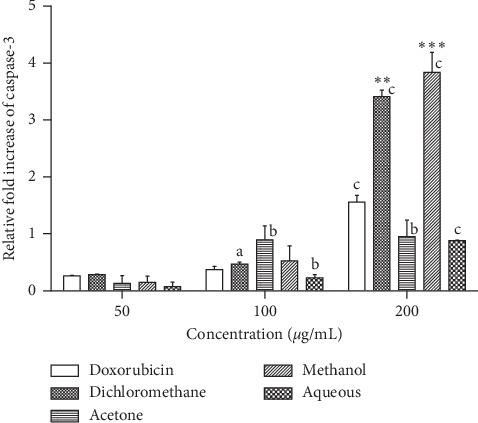
Effects of SAHA extracts and doxorubicin on the activation of caspase-3 in RD cancer cells. Results are mean ± SD (*n* = 2). a represents significant difference (*p* < 0.01), b represents *p* < 0.001, and c represents *p* < 0.0001 when comparing to 50 *μ*g/ml extract or 2 *μ*g/ml doxorubicin. (∗∗) represents *p* < 0.01 and (∗∗∗) represents *p* < 0.001 when compared to 8 *μ*g/mL doxorubicin.

As shown in Figure 3, caspase 3 activity in RD cells significantly increased several folds in a concentration-dependent manner between 50 *μ*g/mL and 200 *μ*g/mL of SAHA extracts and between 2 *μ*g/mL and 8 *μ*g/mL of doxorubicin. For the dichloromethane, methanol and aqueous extracts significant increase in caspase 3 activity (*p* < 0.0001) was observed between 50 *μ*g/mL and 200 *μ*g/mL concentrations of the extract. For the acetone extract significant increase in caspase 3 activity (*p* < 0.001) was observed between 50 *μ*g/mL and 100 *μ*g/mL concentrations of extracts but no significant difference was observed beyond 100 *μ*g/mL concentration of the extract. A significant increase in caspase 3 activity (*p* < 0.0001) was observed between 2 *μ*g/mL and 8 *μ*g/mL concentrations of doxorubicin. Methanol fraction gave the highest fold increase in caspase 3 activity (3.8-fold increase), followed by dichloromethane (3.4-fold increase), acetone (0.9-fold increase), and aqueous (0.8-fold increase) at 200 *μ*g/mL. Doxorubicin gave a fold increase of 1.6 at 8 *μ*g/mL concentration.

**Figure 4:**
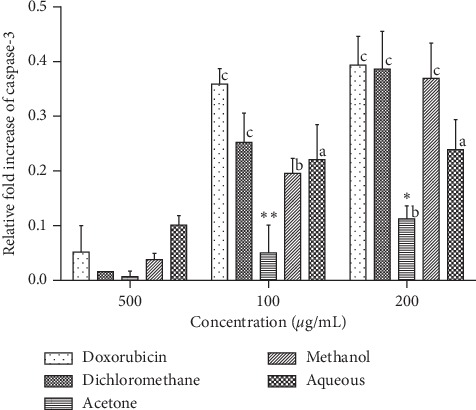
Effects of SAHA extracts and doxorubicin on the activation of caspase-3 in L20B cells. Results are mean ± SD (*n* = 2). a represents significant difference (*p* < 0.01), b represents *p* < 0.001, and c represents *p* < 0.0001 when compared to 50 *μ*g/ml extract or 2 *μ*g/ml doxorubicin.(∗) represents *p* < 0.05 and (∗∗) represents *p* < 0.01 when comparing to 8 *μ*g/mL doxorubicin.

As shown in Figure 4, caspase 3 activity in L20B cells increased in a concentration-dependent manner between 50 *μ*g/mL and 200 *μ*g/mL of SAHA extracts and between 2 *μ*g/mL and 8 *μ*g/mL of doxorubicin. For the dichloromethane, methanol and aqueous extracts significant increase in caspase 3 activity (*p* < 0.0001) was observed between 50 *μ*g/mL and 200 *μ*g/mL concentrations of extracts. A significant increase in the activity of caspase 3 (*p* < 0.001) was observed between 50 *μ*g/mL and 100 *μ*g/mL concentrations of the acetone extract. No significant increase in caspase 3 activity was observed beyond 100 *μ*g/mL concentration of the acetone extract. A significant increase in caspase 3 activity (*p* < 0.0001) was observed between 2 *μ*g/mL and 8 *μ*g/mL concentrations of doxorubicin. However, the fold increase in caspase 3 for L20B cells was significantly lower than that for RD cells. Doxorubicin (8 *μ*g/mL) and dichloromethane fraction (200 *μ*g/mL) gave the highest fold increase of 0.4, followed by methanol (0.36 fold increase), aqueous (0.2 fold increase), and acetone (0.1 fold increase) at 200 *μ*g/mL.

**Figure 5:**
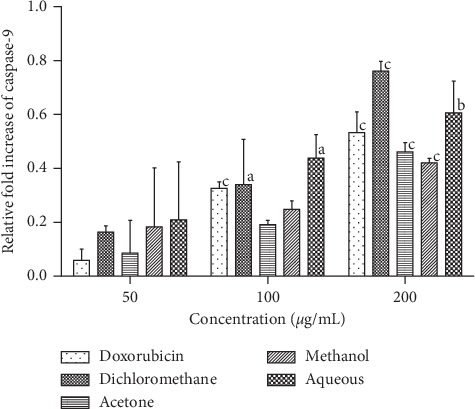
Effects of SAHA extracts and doxorubicin on the activation of caspase-9 in RD cells. Results are mean ± SD (*n* = 2). a represents significant difference (*p* < 0.01), b represents *p* < 0.001, and c represents *p* < 0.0001 when compared to 50 *μ*g/ml extract or 2 *μ*g/ml doxorubicin.

As shown in Figure 5, caspase 9 activity in RD cells increased in a concentration-dependent manner between 50 *μ*g/mL and 200 *μ*g/mL of SAHA extracts and between 2 *μ*g/mL and 8 *μ*g/mL of doxorubicin. For the dichloromethane, acetone, methanol, and aqueous extracts significant increase in caspase 9 activity (*p* < 0.0001) was observed between 50 *μ*g/mL and 200 *μ*g/mL concentrations of extracts. A significant increase in the activity of caspase 9 (*p* < 0.0001) was observed between 2 *μ*g/mL and 4 *μ*g/mL doxorubicin. No significant increase in caspase 9 activity was observed beyond 4 *μ*g/mL concentration of doxorubicin. At 200 *μ*g/mL, dichloromethane fraction gave the highest fold increase of 0.8 followed by aqueous (0.6-fold increase), acetone (0.5-fold increase), and methanol (0.4-fold increase). Doxorubicin gave a fold increase of 0.5 at 8 *μ*g/mL concentration.

**Figure 6:**
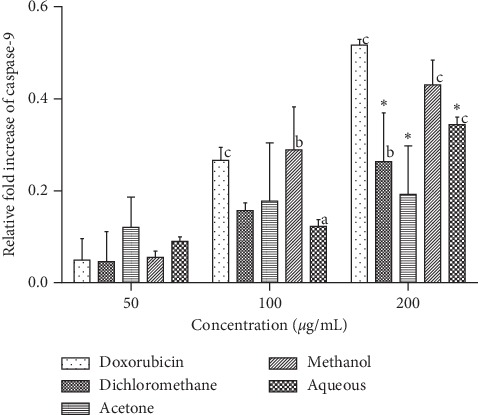
Effects of SAHA extracts and doxorubicin on the activation of caspase-9 in L20B cells. Results are mean ± SD (*n* = 2). a) represents significant difference (*p* < 0.01), b represents *p* < 0.001, and c represents *p* < 0.0001 when compared to 50 *μ*g/ml extract or 2 *μ*g/ml doxorubicin. (∗) represents *p* < 0.05 when compared to 8 *μ*g/mL doxorubicin.

As shown in Figure 6, caspase 9 activity in L20B cells increased in a concentration-dependent manner for some of the SAHA extracts and doxorubicin. For the dichloromethane, methanol and aqueous extracts, significant increase in caspase 9 activity was observed between 50 *μ*g/mL and 200 *μ*g/mL concentrations of the extract. No significant difference was observed in caspase 9 activity at increasing concentrations of the acetone extract. A significant increase in the activity of caspase 9 (*p* < 0.0001) was observed between 2 *μ*g/mL and 4 *μ*g/mL doxorubicin. No significant increase in caspase 9 activity was observed beyond 4 *μ*g/mL concentration of doxorubicin. The fold increase in caspase 9 activity for L20B cells was lower compared to that for RD cells. Methanol extract gave the highest fold increase of 0.4, followed by aqueous (0.3-fold increase), dichloromethane (0.26-fold increase), and acetone (0.2-fold increase) at 200 *μ*g/ml.

### 3.6. Antioxidant Capacity of Treated Cells

#### 3.6.1. Determination of Peroxidase Activity of Treated and Untreated RD and L20B Cells

**Figure 7:**
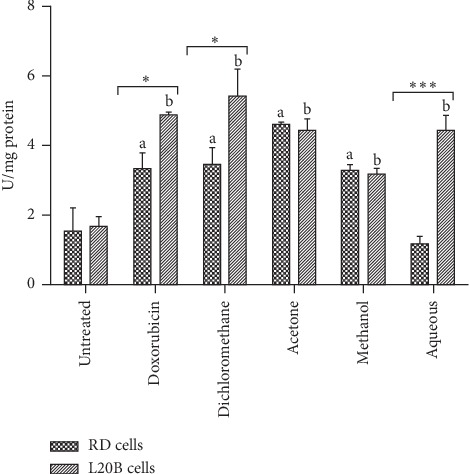
Peroxidase activity in RD and L20B cells after 24 hour treatment with SAHA extracts (200 *μ*g/ml) and doxorubicin (8 *μ*g/ml). Results are mean ± SD (*n* = 2). a and b represent *p* < 0.05 compared to untreated RD and L20B cells, respectively. (∗) represents *p* < 0.05, and (∗∗∗) represents *p* < 0.001 when comparing RD cells to L20B cells.

As shown in Figure 7, the activity of peroxidase was significantly higher in treated RD and L20B cells compared to untreated RD and L20B cells (*p* < 0.05). Treated L20B cells had a higher peroxidase activity compared to the treated RD cells, with dichloromethane fraction giving the highest peroxidase activity of 5.4 U/mg in L20B cells which was significantly higher compared to 3.4 U/mg recorded in RD cells, *p* < 0.05. Doxorubicin gave a significantly higher peroxidase activity of 4.9 U/mg in L20B cells compared to 3.3 U/mg recorded in RD cells, *p* < 0.05. Acetone and aqueous extracts gave higher peroxidase activities of 4.4 U/mg in L20B cells, with the peroxidase activity of aqueous significantly higher in L20B cells compared to RD cells (1.1 U/mg, *p* < 0.001). Methanol fraction recorded the lowest peroxidase activity of 3.3 U/mg in RD cells and 3.2 U/mg in L20B cells.

#### 3.6.2. Determination of Lipid Peroxidation Levels in Treated and Untreated RD and L20B Cells

**Figure 8:**
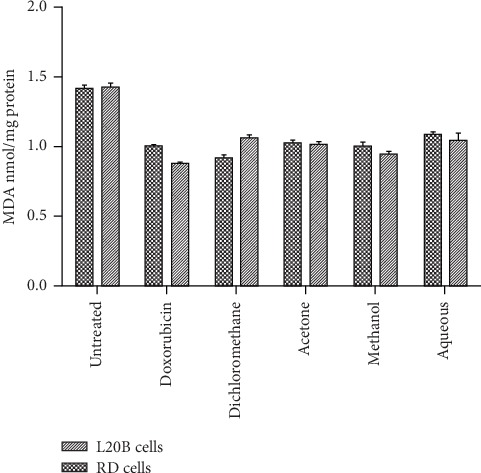
Lipid peroxidation levels in RD and L20B cells after 24-hour treatment with SAHA extracts (200 *μ*g/mL) and doxorubicin (8 *μ*g/mL). Results are mean ± SD (*n* = 3).

As shown in Figure 8, the lipid peroxidation levels in treated RD and L20B cells were significantly lower (*p* < 0.05) than in untreated RD and L20B cells. There was no significant difference in the levels of lipid peroxidation between treated RD cells and treated L20B cells.

## 4. Discussion

Rhabdomyosarcoma is among the world's major public health burden and accounts for high mortality rates in paediatrics [19]. Clinical approaches in the treatment of rhabdomyosarcoma are often associated with high expense, increased toxicity, and adverse side effects. Anticancer compounds that can arrest the cell cycle and induce apoptosis could pave a way for cancer treatment. Phytochemicals such as terpenes, flavonoids, and polyphenols have shown the ability to upregulate apoptotic proteins caspase-3/9 involved in an active form of cell death called apoptosis [20–22].

Phytochemicals extracted from the leaves of *Hibiscus articulatus* and *Sesamum angustifolium* (Oliv.) Engl. were investigated for their combined effects on rhabdomyosarcoma after extraction using different solvents of varying polarity. Of the four extracts that were studied, the dichloromethane extract (IC_50_, 106 *μ*g/mL) recorded the highest cytotoxicity towards RD cells (Table 3). As shown in Table 2, the dichloromethane fraction had a stronger cytotoxic effect on cancerous RD cells compared to the noncancerous L20B cells. The cancer selective character displayed by the dichloromethane fraction has been reported by other workers [23, 24]. The cytotoxic selective character is essential in all potential chemotherapeutic drugs enabling the killing of cancerous cells without harming normal cells.

According to [25, 26], cell death is characterized by morphological changes in a cell. In our study (Figures 6 and 7), untreated RD cells were spindle shaped exhibiting cross-striation, a characteristic of normal muscle cells, and adhered to the surface. Treatment of the cells with IC_50_ of SAHA extracts resulted in morphological changes which included loss of surface adherence, cell shrinkage characterized by round shape, reduced cell density along with cell debris, as shown in Figure 6. The morphological changes observed are characteristic of apoptosis in adherent cells [20]. The results obtained are consistent with those of [27, 28] showing morphological changes characteristic of apoptosis in various cancerous cells, breast (MCF-7 cells), liver (HepG2 cells), lung (NCI-H23), and colon (HT-29). Morphological changes that characterize cell death were not observed in L20B cells treated with SAHA extracts (Figure 7). The treated cells adhered to the surface, retaining the same morphology as those of the untreated cells.

Propidium iodide assay can be used to detect cell death. Propidium iodide stains DNA of cells with damaged membranes. The number of dead cells was expressed as a ratio of total cells present and percentage cell viability expressed as a percentage proportion relative to the untreated cells. The low cell viability obtained at the highest concentration of extracts and doxorubicin in treated RD cells (Table 4) shows that apoptosis induction was more pronounced at higher concentrations of extracts compared to the low concentrations. This could be due to a higher concentration of phytochemicals present at a high concentration of extracts. A similar trend was observed by [18] on Jukart cells after 4 days of treatment with *Triumfetta welwitschii*. As doxorubicin was used as the reference cancer drug, the highest concentration gave a strong apoptosis induction effect in treated RD cells which was significantly higher compared to untreated cells. This shows the effectiveness of doxorubicin as a known anticancer drug against RD cells. The mechanism of cell death induction by doxorubicin is p-53 mediated apoptosis. The apoptotic induction effect of SAHA extracts and doxorubicin were more pronounced in treated RD cells than treated L20B cells (Table 4). In treated L20B cells, the cytotoxicity effect was above 50%, depicting that there were more viable cells as compared to cell death by apoptosis. This could be attributed to the low toxicity of SAHA extracts on L20B cells as compared to RD cells.

The activation of caspase 3 and caspase 9 was evaluated to determine the death pathway induced by SAHA extracts. Caspase 3 is the most important protein involved in the execution pathway of apoptosis. The activation of caspase 3 regulates cell proliferation and reduces the onset of cancers. SAHA extracts showed a concentration-dependent increase in the activation of caspase 3 activity, which may be attributed to the increase in phytochemical content. Phytochemicals have been previously reported for the apoptotic induction effect [29]. The low caspase 3 induction effect of acetone and water extract at 200 *μ*g/mL could be due to the low content of anticancer compounds present. The induction of caspase 3 by SAHA extracts in RD cells was ten times higher than that observed in L20B cells, Figure 4. The activity of caspase 3 in normal cells is highly regulated by a group of proteins collectively known as inhibitors of apoptosis [30]. The level of caspase 3 is usually maintained at low concentrations compared to the level of procaspase 3 [30]. This result is very low apoptosis induction in normal cells. Apoptosis is a protective mechanism that maintains cell homeostasis by removing only rogue cells from tissue milieu [31].

In the intrinsic pathway of apoptosis, the activity of caspase 3 is initiated by initiator caspases such as caspase 9. From the results obtained in Figures 5 and 6 there was no direct relationship in the induction levels of caspase 9 and caspase 3. The activation of caspase 9 was four times lower than that observed in caspase 3 in RD cells. Badmus and others reported a low caspase 9 induction effect of *Holarrhena floribunda* (G. Don) extracts on the breast (MCF-7), colorectal (HT-29), and cervical (HeLa) cancer cells [32]. Our study also observed a low activity of caspase 9 in L20B cells (Figure 6). This was expected as normal cells do not normally undergo apoptosis unless a mutation in DNA arises. Further work is required to determine the actual pathway of cell death by apoptosis.

The SAHA extracts enhanced the activity of the antioxidant enzyme peroxidase, as shown in Figure 7. The peroxidase activity in untreated RD and L20B cells was significantly lower compared to the treated cells. Normal cells are protected by antioxidant enzymes from the toxic effects of high concentrations of reactive oxygen species generated during cellular metabolism. Even though cancer cells generate reactive oxygen species, it has been demonstrated biochemically that antioxidant enzyme levels are low in most animal and human cancers [33].

The activity of antioxidant enzymes in treated L20B cells was significantly higher than that of treated RD cells in dichloromethane and aqueous extracts, and doxorubicin.

Lipid peroxidation is one of the major indications of oxidative stress in cells. One of the byproducts of lipid peroxidation is MDA which is considered to be a mutagen. MDA has been previously used as a biomarker for lipid peroxidation in several *in vitro* studies [34, 35]. High levels of lipid peroxidation indicate high oxidative stress and have been observed in different types of cancers such as breast cancer and lung cancer [36–38]. As shown in Figure 8, SAHA extracts demonstrated the ability to significantly reduce lipid peroxidation in pretreated RD and L20B cells. Untreated RD and L20B cells had significantly higher levels of MDA compared to the treated cells. The high levels of MDA in untreated RD cells are an indication of high oxidative stress in cancerous cells which is a result of depletion of antioxidant enzymes [39]. The phytochemicals in SAHA extracts prevent lipid peroxidation by binding to lipid peroxides. Our findings are in agreement with previously reported results by other workers [40, 41].

## 5. Conclusion

The combined leaf extracts of *Sesamum angustifolium* (Oliv.) Eng. and *Hibiscus articulatus* (SAHA) were selectively cytotoxic to RD cells than L20B cells. The leaf extracts demonstrated a powerful induction of caspase 3 in RD cells than L20B cells. RD and L20B cells pretreated with the combined leaf extracts showed enhanced antioxidant capacity against hydrogen peroxide induced damage and orthophosphoric acid induced lipid peroxidation. Further in vivo studies are being done to make it clear whether the combined leaf extracts could be proposed as a natural agent for the prevention and treatment of rhabdomyosarcoma.

## Figures and Tables

**Table 1:** Yield of *Sesamum angustifolium* (Oliv.) Engl. and *Hibiscus articulatus* crude leaf extracts.

Plant species	Yield (%)			
	Dichloromethane	Acetone	Methanol	Water
*Sesamum angustifolium* (Oliv.) Engl.	7.1 ± 3.1	16.7 ± 1.4	18.5 ± 1.1	23.1 ± 3.0
*Hibiscus articulatus*	7.1 ± 2.0	16.9 ± 2.3	20.5 ± 1.0	23.4 ± 3.2

Results are expressed as mean ± SD (*n* = 3).

**Table 2:** Cell viability of RD and L20B cells after 48-hour treatment with SAHA extracts and doxorubicin.

SAHA extract	Concentration (*μ*g/mL)	Cell viability (%)	
		RD cells	L20B cells
Dichloromethane	200	39.5 ± 5.1^a^	64.5 ± 4.1^*∗*^
Dichloromethane	100	45.4 ± 9.7^a^	71.4 ± 6.0^*∗*^
Dichloromethane	50	47.1 ± 5.7^a^	72.0 ± 8.6^*∗*^
Dichloromethane	25	63.1 ± 4.6	78.2 ± 5.8^*∗*^
Dichloromethane	12.5	77.0 ± 7.3	85.6 ± 2.1
Acetone	200	43.4 ± 4.9^a^	68.4 ± 7.2^*∗*^
Acetone	100	61.6 ± 3.5	72.3 ± 9.2^*∗*^
Acetone	50	73.3 ± 7.3	74.4 ± 12.1
Acetone	25	79.2 ± 5.6	74.3 ± 10.2
Acetone	12.5	84.3 ± 5.5	92.9 ± 5.7
Methanol	200	40.0 ± 6.2^a^	79.3 ± 4.4^*∗∗∗*^
Methanol	100	48.3 ± 5.6^a^	80.6 ± 8.9^*∗*^
Methanol	50	64.1 ± 3.5	86.0 ± 5.0^*∗*^
Methanol	25	63.8 ± 6.3	86.2 ± 3.0^*∗*^
Methanol	12.5	76.6 ± 10.1	89.7 ± 4.8
Aqueous	200	41.4 ± 3.0^a^	62.5 ± 1.8^*∗*^
Aqueous	100	50.7 ± 1.6^a^	63.2 ± 3.8
Aqueous	50	58.9 ± 5.8^a^	66.9 ± 4.5
Aqueous	25	74.3 ± 4.0	70.3 ± 2.7
Aqueous	12.5	83.6 ± 1.3	79.4 ± 8.6
Doxorubicin	8	30.5 ± 2.4^a^	59.2 ± 7.5^b*∗*^
Doxorubicin	4	34.2 ± 4.1^a^	71.0 ± 9.4^*∗∗∗*^
Doxorubicin	2	40.1 ± 5.4^a^	73.9 ± 6.1^*∗∗∗*^
Doxorubicin	1	44.4 ± 6.8^a^	77.5 ± 10.0^*∗∗∗*^
Doxorubicin	0.5	57.2 ± 11.1^a^	81.9 ± 5.3^*∗*^

The number of live negative control cells (0 *μ*g/mL) at 48 h was used as 100% viability, so all cells were expressed as a percentage of them. Results are expressed as mean ± SD (*n* = 3). a and b represents significant difference (*p* < 0.001), compared to untreated RD and L20B cells, respectively. ^*∗*^ indicates *p* < 0.05 and ^*∗∗∗*^ indicates *p* < 0.0001 when comparing RD cells to L20B cells.

**Table 3:** IC_50_ values for treated RD cells.

Extract	RD cells IC_50_ (*μ*g/mL)
Doxorubicin	0.8
Dichloromethane	106
Methanol	122
Aqueous	129
Acetone	158

**Table 4:** Propidium iodide assay to determine apoptosis induction effect of SAHA extracts and doxorubicin in RD and L20B cells after 48 hour treatment.

Extract	Concentration (*μ*g/mL)	Viability (*T*/*C*) (%)	
		RD cells	L20B cells
Doxorubicin	8	49.7 ± 5.7	57.7 ± 17.2
	2	68.2 ± 8.1	68.2 ± 14.7
Dichloromethane	200	55.1 ± 3.2	66.7 ± 14.4^*∗*^
	50	63.5 ± 12.3	60.0 ± 10.1
Acetone	200	60.9 ± 1.0	78.3 ± 33.6^*∗*^
	50	66.6 ± 8.6	60.5 ± 4.5
Methanol	200	48.4 ± 2.7	65.1 ± 10.8^*∗*^
	50	50.9 ± 7.8	55.6 ± 13.0
Aqueous	200	72.1 ± 3.2	85.9 ± 13.4^*∗*^
	50	93.0 ± 10.6	83.4 ± 10.0^*∗*^

The number of live negative control cells (0 *μ*g/mL) at 48 h was used as 100% viability, so all cells were expressed as a percentage of them. Results are mean ± SD of two replicates (*n* = 2). ^*∗*^ indicates *p* < 0.05 comparing treated RD cells to treated L20B cells of similar concentration.

## Data Availability

The data used to support the findings of this study are available from the corresponding author upon request.
